# Relationship between child opportunity index and body mass index *z*-score: a mixed-effects analysis with data from a lifestyle intervention with Hispanic children

**DOI:** 10.3389/fradm.2025.1547910

**Published:** 2025-05-12

**Authors:** Christian E. Vazquez, Bethany Wood, Swasati Handique, Yuanyuan Liang, Zenong Yin, Deborah Parra-Medina

**Affiliations:** 1 School of Social Work, The University of Texas at Arlington, Arlington, TX, United States,; 2 Department of Epidemiology and Public Health, School of Medicine, University of Maryland, Baltimore, MD, United States,; 3 Department of Public Health, The University of Texas at San Antonio, San Antonio, TX, United States,; 4 Center for Health Equity, University of Colorado Anschutz Medical Campus, Aurora, CO, United States

**Keywords:** environmental factors, body mass index *z*-score, intervention, Hispanic, multilevel model

## Abstract

**Background:**

The Childhood Opportunity Index (COI) is a relatively new measure for assessing opportunity across education, health and environment, and socioeconomic context. Research indicates that higher COI is associated with lower obesity risk; however, existing research offers no evidence for differences, or lack thereof, across racial/ethnic groups. The larger body of research on the relationship between neighborhood environments and obesity risk among Hispanic children with low-income between 5 and 11-year-olds is limited. The study aims to further explore the relationship between neighborhood opportunities, measured by the COI, and children’s body mass index *z*-scores (BMIz), adjusted for age and sex.

**Materials and methods:**

The data are from a sample of Hispanic child-parent dyads (*n* = 253) who participated in a 1-year family lifestyle intervention. A linear mixed-effects model was fitted, with BMIz as the dependent variable, COI categorized into four levels, time, parent BMI, family income, adult education, child age, child sex, calories, language spoken in the household, physical activity, group condition, and a time*group condition interaction. Predicted probabilities were also produced.

**Results:**

After adjusting for covariates, children in the second (*β* = −.15, 95% CI = −0.27, −0.03), third (*β* = −.19, 95% CI = −0.31, −0.06), and fourth (*β* = −.15, 95% CI = −0.28, −0.02) quartiles of the COI quartiles had significantly lower BMIz compared to those in the first (lowest) COI quartile. Predicted probabilities show the different predictive margins of BMIz at each time point for each quartile compared to the first quartile.

**Conclusions:**

All the higher COI levels were linked to healthier weight status compared to the lowest COI level, though the pattern was not linear for any of the observed associations. Further investigation into the impact of different COI levels may be warranted to assess each quartile’s impact against each other, which was outside the scope of the current study. Results also provide evidence for potentially strengthening intervention supports for those at the lowest COI level, respective to those from all other COI levels.

## Introduction

Childhood obesity remains a significant public health challenge in the United States (U.S.). Hispanic youth aged 2–19 years exhibit a higher obesity prevalence (26.2%) compared to non-Hispanic White (16.6%), non-Hispanic Black (24.8%), and non-Hispanic Asian youth (9.0%) (1). These rates underscore the disparities in obesity prevalent among different racial/ethnic groups, with Hispanic and Black children bearing a disproportionate burden. Hispanic and Black families are more likely to live in disadvantaged obesogenic neighborhoods with increased risk for early onset of obesity compared to their White peers (2, 3). Hispanic children in south Texas have the highest rates of childhood obesity among all their peers and greatly exceed national averages (4). Prior studies have found household characteristics and social environment factors significantly influence the body mass index (BMI) of Hispanic children (5); though, the research on the relationship between neighborhood environments and obesity risk among low-income Hispanic children between 5 and 11-years-old is limited (6). The combination of this evidence, or lack thereof, warrants further study on the relationship between neighborhood opportunities for healthy development and childhood obesity among Hispanic children. The current study focuses on further understanding how neighborhood opportunities, via the Child Opportunity Index (COI), are related to childhood BMI among Hispanic children who participated in a family lifestyle intervention over 1 year.

In the U.S., several factors are generally associated with obesity in youth. For example, school-aged boys have a higher obesity rate (20.4%) than girls (16.3%) (7). Dietary habits (8), sedentary behavior (9, 10), parental education level (11), and low socioeconomic status (SES) (7) are all also related to the higher prevalence of obesity among Hispanic children. Parental weight status is also related to childhood obesity (12). Along with this, cultural factors too play a role, such as Hispanic parents underestimating their child’s weight status, not believing it is a problem, or being adverse to healthy behaviors including physical activity (13–16).

Existing literature on childhood obesity primarily relies on cross-sectional individual level data to establish associations between sociodemographic factors and behaviors (17). The sparse literature using neighborhood level data provides some insights for the current study. Studies have shown that residing in high-poverty or high-crime neighborhoods is associated with an increased risk of high BMI or childhood obesity (18). Another study by Theall et al. (19) identified an association between neighborhood violent crime and negative outcomes for obesity-related outcomes like BMI, overweight/obesity, and waist circumference. Research has also found that broader factors such as higher average SES of a child’s school is linked to lower BMIz, among pre-kindergarten students (20). Similarly, research shows that children from low-income communities exhibit lower levels of physical activity, poor dietary habits, with increased consumption of fried food and sugary beverages along with spending more time in sedentary activities (21). Finally, a meta-analysis of 58 randomized controlled trials (RCTs) in the US showed that interventions with a higher number of young children of racial/ethnic minority or lower socioeconomic status were less effective in lowering obesity measures signifying the influence of community context on the uptake of evidence-based obesity prevention strategies (22). All these factors are associated with higher BMI and obesity. The literature highlights the importance of assessing neighborhood factors that influence the level of protection against childhood obesity.

The COI is a publicly available cumulative indicator of the many positive and negative attributes of neighborhood conditions and resources that greatly influence healthy child development (23). The COI index is built using 44 indicators that span over three domains: education, health and environment, and socioeconomic context and 14 sub-domains (24). The indicators are associated with children’s health and economic outcomes. The COI is a multidimensional measure which incorporates data from the US Census Bureau’s American Community Survey and other sources capturing inequities in distribution of opportunity across neighborhoods (23). The COI is unique from other census tract-based opportunity indices as it utilizes a wide range of novel negative factors such as exposure to toxic waste, school poverty, and supportive factors such as access to nutritious food and green spaces. Studies have linked high COI scores with decreased risk of acute care visits (25), asthma-related hospitalizations (26), and improved preventive care metrics in pediatric primary care (23). Additionally, research suggests associations between higher COI and lower risk of obesity and cardiometabolic risk in children (17). Aris et al. (17) found that exposure to a high COI score at birth significantly affects mean BMI and obesity risk compared to exposure at later life stages. The COI is a relatively newer index and evidence suggests it can be a valuable tool for identifying children at risk of progressing towards a trajectory of high BMI as it incorporates various neighborhood characteristics relevant to children’s health. The current study focuses on the relationship between baseline COI and obesity over a 1-year period. Based on existing literature, it is expected that lower COI scores will be related to higher BMI; however, previous studies have not examined this with BMI over time and with an all-Hispanic sample of 5- to 11-year-olds. An additional justification for this study is that it is not clear if COI is related to BMI for Hispanic children in the same way as children in samples from other studies above. It may be the case that Hispanic children, particularly those in south Texas, who live in a neighborhood with others that share contextual factors (i.e., similar COI) may be protected against adverse effects on weight status (27, 28). This would result in no differences by COI. In addition to adding to the limited literature on COI, the current study adds rigor with the rich individual- and family-level control variables to more effectively isolate the community effect.

## Materials and methods

### Data

The current study is a secondary analysis of data extracted from a family-based obesity management study, “Health4Kids” (H4K), in Hispanic children ages 5–11 years and their parents (*N* = 253 dyads). Considering the strong cultural value of familism within Hispanic communities, where family takes central importance, family-focused obesity prevention strategies may be particularly effective (29). This randomized controlled trial aimed to examine the efficacy of “Health4Kids” (H4K), a family-focused intervention promoting healthy eating and physical activity for Hispanic children aged 5–11 years with overweight/obesity. The program utilized family-centered behavioral counseling, text messages, and newsletters to encourage healthier eating habits and increased physical activity among participants. The study was conducted in south-central Texas. The families were randomized into the standard care group (*n* = 128) or the H4K treatment group (*n* = 125). The participants completed assessments at baseline (*n* = 253), 1-month (*n* = 179), 6-month (*n* = 184), and 12-month (*n* = 153). Activities and assessments were available in Spanish and English both oral and written. More information about the design, data, IRB approval, and materials can be found elsewhere (NCT02343367).

### Variables

In this paper, the dependent variable was child BMI z-score for age and sex (BMIz), a continuous variable measured at four time points (baseline, 1-month, 6-month, and 12-month). The primary independent variable was the COI measured at baseline. We use baseline COI because COI would not change over a 1-year period unless a family moved, and COI is only calculated by external researchers every 5 or so years. The COI ranges from 1 to 100 and is used as an indicator of the amount of opportunity that children have in their neighborhoods in their social and economic domains (30). The COI has been used in other studies as a predictor or covariate of child BMI (17). In the current study, the COI national adjusted z-score (COIz) is used. COIz, as a continuous variable, did not have a strong linear relationship with BMIz. The path was more of an upside-down checkmark as opposed to a straight line so it was divided into quartiles for the analyses to address the non-linearity problem. The four quartiles represent very low, low, high, and very high as has been used in previous studies (17). The study data and COI variable were linked via geocoding. The families represented 14 census tracts.

The following section provides a brief explanation for the inclusion of some of the control variables. Variables for child age, sex, physical activity, diet, and parent BMI, income, education were included as they are consistently associated with BMI differences in childhood (28, 31). Additionally, language spoken in the home was relevant given the sample are south Texan Hispanics where this is a high prevalence of Spanish speakers. Language spoken in the home has been included in several childhood obesity studies with mixed findings as language can be tied to acculturation, and can be both a protective or risk factor for healthy behaviors (32–34).

Other covariates considered in the analysis included time, a categorical variable representing the wave of data collection [0 = baseline, 1 = 1 month, 6 = 6 months, 12 = 12 months] and child age ranging from 5 to 11 years old, held fixed at baseline to avoid multicollinearity with time. Child demographics also included binary child sex [0 = female (reference), 1 = male], child time spent in moderate-to-vigorous physical activity (MVPA; minutes/day), a continuous variable using an ActiGraph triaxial accelerometer (GX3). Each participant wore the monitor for 7 days and 6 days of data were collected, accelerometer counts were recorded in 60-s time increments (35). Ranges were 0–5,725+, with inactivity (0–99 counts/min), light (100–759 counts/min), moderate-intensity lifestyle activities (760–5,724 counts/min), moderate-intensity walking activities (1,952–5,724 counts/min), and vigorous intensities (5,725 or more counts/min) (35). Additionally, the average daily calories for each child were measured using the Block Food Screeners for Ages 2–17, which included the average daily calories for each child as a continuous covariate (36). The validated screener was designed to be self-administered by children with the assistance of parent or caregiver, as needed.

Parental factors included parent BMI, a continuous variable, parent education, a categorical variable [0 = high school education or less (reference), 1 = some college, and 2 = four-year college degree or higher], and parent income, a categorical variable of income levels [0 = <$10,000 (reference), 1 = $10,001–$15,000, 2 = $15,001–$20,000, 3 = $20,001–$25,000, 4 = $25,001–$35,000, 5 = $35,001–$50,000, 6 = ≥$50,001, and 7 = Missing]. Spanish spoken at home was coded as a binary variable [0 = households speaking only English (reference), 1 = households where Spanish is spoken, either mixed with English or exclusively]. Finally, treatment group was coded as a binary variable [0 = standard care (reference), 1 = group condition].

### Analyses

Data was cleaned, prepared, and analyzed using STATA/SE version 17 (37). For each wave, we examined sample size and descriptive statistics across the total sample and for COI quartiles. To assess baseline characteristics differences across COI quartiles groups, ANOVA, Chi-square test or Fisher’s exact were used. Full information maximum likelihood (FIML) method was used to handle missing data in the outcome variable, BMIz. This approach was chosen because the amount of missing data was relatively low. Specifically, all covariates had less than 10% missing data, with the exception of parent income. For parent income, a missing category was added as the final category in the nominal variable. To test for changes in children’s BMIz, we used linear growth curve models constructed in a sequential bottom-up approach: An unconditional growth model, followed by a combined fixed effects model, random slopes testing, interaction testing, and evaluation of different within-subject covariance matrices.

The unconditional growth model was first estimated using time as a continuous variable with a quadratic term, but due to non-significance and worsened model fit, it was ultimately used as a categorical variable. Time points and child ID were treated as random effects to obtain random intercepts for child participants and estimate the random slope of time for each child ID. For the next two steps, COI quartiles and covariates were incorporated into the model as fixed effect individual-level predictors. To avoid multicollinearity with the time variable, child age at baseline was used as a fixed effect. Additionally, MVPA scale, calories, and adult BMI were also grand mean centered.

Several interactions were tested, including COI*Group condition, COI*MVPA scale, and COI*Time, but were excluded from the final model due to non-significance and worsened fit. The Time*Group condition interaction, although non-significant, was retained on a theoretical basis.

This process culminated in two final models: an unconditional growth model and a linear mixed-effects model with a random slope. The latter included the Time*Group condition interaction term, a random slope for time within child participants, and an independent covariance structure. Except for the unconditional growth model, all covariates were included in the models.

Model fit was assessed by comparing improvements in −2LL, AIC, and BIC indices relative to previously nested models, ensuring a comprehensive evaluation of the model’s performance and appropriateness for the data. Models were fitted using Stata’s mixed command with default REML estimation.

## Results

The final sample was *n* = 253 children. Child baseline BMI ranged from 16.52 to 34.25 (between the 73rd and 99th percentile), and baseline BMIz ranged from 0.62 to 2.68. The mean BMIz ranged from 1.74 to 1.80 across waves. The mean child BMI in each wave ranged from 22.73 to 23.86 (the 95.00th and 95.50th percentiles, respectively), with a 39.5% attrition reduction in child BMI in the final wave of data collection. Just over half of both the treatment and control groups were comprised of female children. The most common family income bracket was $10,001–$15,000. Over 60% of the sample had a Spanish speaking household or a mixed Spanish and English-speaking household. As far as parental education level, approximately 40% of the children’s parents had some college/technical school, 30% had a high school or less education, and 13% had a four-year degree or more. Just over 49% of the sample were in the H4K treatment group. The mean average daily calories for children were 1,145 kcal (SD = 458.03) and the mean MVPA score was 252.60 (SD = 153.73). The COIz ranged from −0.08 to 0.027. This represented families with very low to very high COI. All sample demographics by COI quartile are reported in Table 1. Only parent education level was significantly different *χ*^2^ (6, *N* = 224) = 15.92, *p* = 0.014 at baseline across the COI quartiles. There were more parents with a four-year degree or more in the 3rd and 4th quartiles.

### Unconditional model

The unconditional model (Table 2) showed that, on average, BMIz was significantly higher at 1-month compared to baseline (*β* = .05, 95% CI = 0.03, 0.07). No significant differences were observed between baseline and the other time points. This model also revealed a high intraclass correlation of 0.89, as expected.

### Final model

**FIGURE 1:**
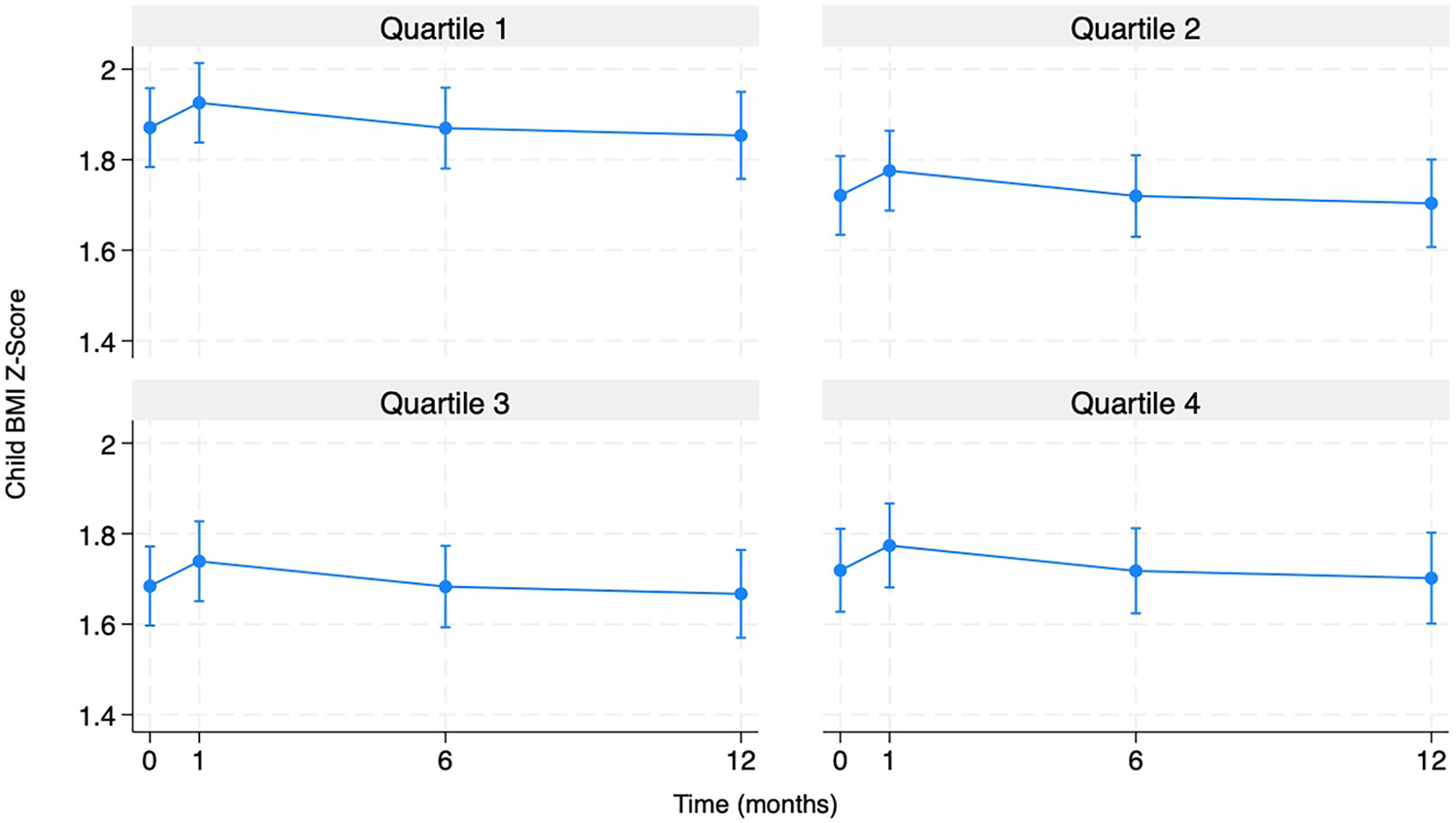
Predicted child BMI *z*-scores by child opportunity index (COI) quartiles over time (*n* = 253).

The final model included the following variables: categorical time, COI (in quartiles), parent BMI, family income, adult education, child age, child sex, average daily calories, language spoken in the household, MVPA, group condition, and Time*Group condition (Table 2). Among children in the control group, wave 1 BMIz differed significantly compared to baseline in that wave 1 BMIz was higher at wave 1 compared to baseline (*β* = .08, 95% CI = 0.04, 0.11). The coefficient for the intervention group was *β* = .04 and this was statistically significant compared to the control group (95% CI = −0.10, 0.01). The other waves were not significantly different from baseline. Compared to children in the first (lowest) COI quartile, being in the second (*β* = −.15, 95% CI = −0.27, −0.03), the third (*β* = −.19, 95% CI = −0.31, −0.06), and the fourth (*β* = −.15, 95% CI = −0.28, −0.02) quartiles were associated with lower BMIz. Generally, each higher neighborhood opportunity level was associated with lower BMIz values when compared to the lowest opportunity level. Figure 1 shows the model-based estimated margins of BMIz at each time point by COI quartiles. Being male was significantly associated with a higher BMIz in children (*β* = .18, 95% CI = 0.09, 0.28). An increase in child age was significantly associated with an increase in BMIz (*β* = .03, 95% CI = 0.00, 0.06). Additionally, higher adult BMI was positively associated with an increase in child BMIz (*β* = .01, 95% CI = 0.00, 0.02). Parents with some college education had a higher BMIz compared to those with a high school or less level of education (*β* = .11, 95% CI = 0.01, 0.21). Regarding income, only the “$20,001–$25,000” (*β* = 0.23, 95% CI = 0.06, 0.39) and “≥$50,001” (*β* = .22, 95% CI = 0.02, 0.42) brackets were significantly associated with increased BMIz in children compared to the reference group of less than $10,000. No significant effects were found for the MVPA scale, average daily calories, or language spoken in household.

## Discussion

The current study focused on further understanding how neighborhood opportunities, via the COI, are related to childhood weight status among Hispanic children in south-central Texas who participated in a family lifestyle intervention over 1 year. Notably, though not all individual children fell into this category, the mean BMIz for this sample ranged in the obese category across the waves, which was expected due to criteria and focus of the larger study. This sample also represented a diverse range of COI levels. The current study findings appear to align with COI and BMI studies with non-Hispanic samples that do not necessarily represent a high-risk and diverse COI population (17). Compared to children in the lowest COI quartile, being in the second, third, or fourth quartile was associated with lower BMIz. Though these findings are not novel, Figure 1 shows the nuance of this finding as the predictive margins for BMIz at each time point follow different patterns for each quartile relationship observed. Follow up studies can further investigate what about each level of opportunity [i.e., what value(s) of COI] may be associated with risk or protection against poorer weight status.

The main effects align with some existing literature on global patterns, suggesting that being male (38) and higher parent BMI are associated with increased BMIz (12). Age as a predictor of increased BMI is not always conclusive, though the significant finding from the current study aligns with the literature for children of this age and developmental period (38). The literature typically finds higher parent education and higher income to be associated with lower BMI; however, the inverse was found here. This is not surprising when one considers the overall average level of education and income for this group, which was low, and the overall relationship between socioeconomic status and childhood obesity. Evidence exists to support that when comparing groups of similar SES, slight differences appear such that those at lower levels of what might be considered poverty fare better than those at the higher end or those in higher SES neighborhoods who are at the higher end of wealth fare better than those at lower end of what is considered to be wealthy even though they are in the same neighborhood (27, 28). This maps on to the findings from Figure 1. For example, if a household is significantly economically disadvantaged, they may benefit from safety net programs that provide needed resources but if the household is just over the eligibility threshold, then they may not benefit from extra support though there may be a clear need for it. However, the data does not have the information to capture and compare varying levels and if they match eligibility levels. Further, there may be a protective factor of feeling like you belong in your neighborhood, though that was not measured in the current study. No statistically significant effects were found for other factors that have been found in the literature such as physical activity, child calorie intake, and language spoken in household.

The current study also did not find any BMIz differences between treatment groups. It should be acknowledged that this is not uncommon with an intervention sample of this size. Further, the descriptive statistics did indicate substantial variability in BMIz at the baseline across different groups so any potential differences may have been attributed to other statistically significant factors above. Additionally, Wave 1 BMIz differed significantly compared to baseline in that wave 1 BMIz was higher at wave 1 compared to baseline but there was no difference throughout the rest of the time points. It could be the case that the sample gained weight for any number of reasons but looking at the trends of the data, the intervention may have been working for certain groups though perhaps a longer follow up or intervention period would have produced significant results. The analysis also did not detect interaction effects for COI*Group condition, COI*physical activity, and Time*Group condition. This may be attributed to power, but further investigation is warranted given what is seen in Figure 1.

The lack of a treatment effect finding is consistent with the findings of Yin et al. (39), which emphasized the need for multi-level and multi-behavioral approaches to effectively address obesity among young children from low-income families. Though the current study addressed various factors, the results may indicate the importance of considering even broader social determinants of health (SDOH) in obesity-based interventions (40, 41). Still, the current study findings suggest that intervening on the areas measured by the COI could be an effective strategy for reducing obesity risk among Hispanic children, thereby supporting the call for multi-level interventions and changes in SDOH.

## Limitations

As with any study using BMI as an outcome measure there are limitations in what the real-life implications are given the known challenges with this measure (42). However, the use of standardized measurement and use of *z*-score help strengthen the rigor of the variable. Though the findings align with other published studies (17), the current findings may only hold true among Hispanic 5- to 11-year-olds in south-central Texas. As noted above, there might not have been enough power in this study to detect certain difference at the *p* < .05 level. This was a diverse but relatively low COI sample and including another group with starker differences could elucidate more information about varying levels of COI, though that was outside the scope of the parent study. Notably, while not all children in the sample had baseline BMIz scores within the range or overweight or obese, all children participated in a family lifestyle intervention aimed at improving health lifestyle behaviors such as physical activity and dietary habits. Still, the analysis controlled for treatment and time to account for participation in the intervention.

## Conclusions

The research on the relationship between neighborhood environments and obesity risk among low-income Hispanic children between 5 and 11-years-old continues to be limited. The current study suggests that there are potential healthy weight differences across levels of child opportunity, using a robust measure of the environmental context, though further research is needed to understand causal relationships. Understanding which indicators within the COI to intervene on for the largest impact would be helpful to further refine family and multi-level interventions. Finally, though the patterns of low opportunity remain linked to unhealthy weight status, there are nuances to be examined to further understand what protective factors can be drawn out of each COI group to utilize a strengths-based approach to good health.

## Figures and Tables

**TABLE 1:** Sample means, frequencies, and characteristics by child opportunity index (COI) quartiles (*n* = 253).

Variables	Total	1st quartile	2nd quartile	3rd quartile	4th quartile
	Mean (SE)	Mean (SE)	Mean (SE)	Mean (SE)	Mean (SE)
Child BMI *z*-score at wave 0*	1.74 (0.35)	1.82 (0.35)	1.75 (0.37)	1.69 (0.32)	1.72 (0.36)
Child BMI *z*-score at wave 1	1.80 (0.35)	1.88 (0.34)	1.76 (0.36)	1.77 (0.33)	1.80 (0.39)
Child BMI *z*-score at wave 6	1.75 (0.40)	1.82 (0.37)	1.76 (0.40)	1.72 (0.37)	1.71 (0.45)
Child BMI *z*-score at wave 12	1.74 (0.41)	1.77 (0.37)	1.74 (0.40)	1.64 (0.42)	1.82 (0.43)
Overall child BMI score**	22.73 (3.21)	23.44 (3.16)	22.90 (3.41)	22.24 (2.81)	22.27 (3.34)
Adult BMI***^,^****	33.19 (7.00)	33.75 (7.70)	33.41 (6.61)	33.24 (7.50)	32.51 (7.39)
Child age, year***^,^*****	8.74 (1.67)	8.94 (1.58)	8.86 (1.64)	8.69 (1.71)	8.45 (1.82)
MVPA scale***^,^******	252.60 (153.73)	232.49 (145.78)	270.25 (170.91)	231.78 (128.79)	277.04 (163.72)
Child average daily calories***^,^*******	1,145.38 (458.03)	1,209.60 (470.02)	1,087.79 (506.74)	1,070.18 (353.04)	1,220.02 (480.84)
**Overall *N* (%)**					
		66 (26.09)	65 (25.69)	64 (25.30)	58 (22.92)
**Group condition** ********					
Treatment group	125 (49.41)	34 (48.48)	32 (50.77)	30 (46.88)	29 (50.00)
Control group	128 (50.60)	34 (51.52)	33 (49.23)	34 (53.12)	29 (50.00)
**Household language** *********					
Only English	74 (29.25)	22 (33.33)	18 (27.69)	20 (31.25)	14 (24.14)
Only Spanish/Mixed Spanish and English	160 (63.24)	37 (56.06)	42 (64.62)	39 (60.94)	42 (72.41)
**Adult education level** **********					
High school or less	86 (33.99)	22 (33.33)	27 (41.54)	18 (28.12)	19 (32.76)
Some college/technical school	105 (41.50)	31 (46.97)	28 (43.08)	26 (40.62)	20 (34.48)
Four year college degree or more	33 (13.04)	5 (7.58)	2 (3.08)	13 (20.31)	13 (22.41)
**Child sex** ***********					
Male	125 (49.41)	33 (50.00)	26 (40.00)	37 (57.81)	29 (50.00)
Female	128 (50.59)	33 (50.00)	39 (60.00)	27 (42.19)	29 (50.00)
**Family income** ************					
<$10,000	35 (13.83)	12 (18.18)	6 (9.23)	9 (14.06)	8 (13.79)
$10,001–$15,000	43 (17.00)	12 (18.18)	14 (21.54)	11 (17.19)	6 (10.34)
$15,001–$20,000	26 (10.28)	6 (9.09)	8 (12.31)	7 (10.94)	5 (8.62)
$20,001–$25,000	33 (13.04)	7 (10.61)	9 (13.85)	8 (12.50)	9 (15.52)
$25,001–$35,000	33 (13.04)	8 (12.12)	10 (15.38)	8 (12.50)	7 (12.07)
$35,001–$50,000	26 (10.28)	4 (6.06)	8 (12.31)	8 (12.50)	6 (10.34)
$50,001 or more	17 (6.72)	5 (7.58)	1 (1.54)	4 (6.25)	7 (12.07)
Missing	40 (15.81)	12 (18.18)	9 (13.85)	9 (14.06)	10 (17.24)

Footnotes provided here represent *p*-values for analysis of variance (ANOVA) or Pearson’s chi-squared results for each variable by quartiles.

* *p* = .19.

** *p* = .10.

*** Indicates a grand mean centered variable.

**** *p* = .82.

***** *p* = 0.38.

****** *p* = .24.

******* *p* = .14.

******** *p* = .96.

********* *p* = .53.

********** *p* = .01.

*********** *p* = .25.

************ *p* = .90.

**TABLE 2:** Growth curve models with fixed effects, random effects, and interaction effects on child BMI *z*-score (*n* = 253).

Fixed effects	Unconditional growth model		Final model	
	Estimate [95% Confidence interval]	*p*-value	Estimate [95% Confidence interval]	*p*-value
Intercept	1.74 [1.70, 1.79]	*p* < .001	1.28 [0.97, 1.60]	*p* <.001
**Time**				
0	–		–	
1	0.05 [0.03, 0.07]	*p* < .001	0.08 [0.04, 0.11]	*p* <.001
6	0.01 [−0.01, 0.04]	*p* = 0.32	−0.01 [−0.05, 0.04]	*p* = 0.79
12	−0.00 [−0.04, 0.04]	*p* = 0.97	−0.02 [−0.09, 0.04]	*p* = 0.46
**Child opportunity index (COI)**				
1st quartile			–	
2nd quartile			−0.15 [−0.27, −0.03]	*p* = 0.02
3rd quartile			−0.19 [−0.31, −0.06]	*p* = 0.003
4th quartile			−0.15 [−0.28, −0.02]	*p* = 0.02
Adult BMI^a^			0.01 [0.00, 0.02]	*p* = 0.002
Child Age^a^			0.03 [0.00, 0.06]	*p* = 0.03
MVPA Scale^a^			−0.00 [−0.00, 0.00]	*p* = 0.22
Child average daily calories^a^			−0.00 [−0.00, 0.00]	*p* = 0.13
**Household language**				
Only English			–	
Only Spanish/Mixed Spanish and English			0.02 [−0.08, 0.11]	*p* = 0.72
**Adult education level**				
High school of less			–	
Some college/technical school			0.11 [0.01, 0.21]	*p* = 0.03
Four year college degree or more			0.09 [−0.05, 0.24]	*p* = 0.21
**Child sex**				
Female			–	
Male			0.18 [0.09, 0.28]	*p* <.001
**Group condition**				
Control group			–	
Treatment group			0.07 [−0.02, 0.16]	*p* = 0.15
**Family income**				
<$10,000			–	
$10,001–$15,000			0.09 [−0.06, 0.25]	*p* = 0.22
$15,001–$20,000			0.06 [−0.11, 0.23]	*p* = 0.51
$20,001–$25,000			0.23 [0.06, 0.39]	*p* = 0.007
$25,001–$35,000			0.15 [−0.01, 0.32]	*p* = 0.07
$35,001–$50,000			0.04 [−0.14, 0.21]	*p* = 0.69
$50,001 or more			0.22 [0.02, 0.42]	*p* = 0.03
Missing			0.12 [−0.07, 0.31]	*p* = 0.22
Time *Group condition				
1			−0.04 [−0.10, 0.01]	*p* = 0.11
6			0.01 [−0.05, 0.07]	*p* = 0.76
12			0.02 [−0.08, 0.11]	*p* = 0.75
**Random parts**				
μ_0j_	0.00 [0.00, 0.00]	*p* < .001	0.00 [0.00, 0.00]	*p* <.001
μ_1j_	0.11 [0.09, 0.13]	*p* < .001	0.09 [0.07, 0.11]	*p* <.001
μ_ij_	0.01 [0.01, 0.02]	*p* < .001	0.01 [0.01, 0.02]	*p* <.001
−2LL deviance	48.91		62.45	
AIC	−83.81		−72.22	
BIC	−51.29		55.05	
ICC	0.8871			

a Indicates a grand mean centered variable.

## Data Availability

The datasets presented in this study can be found in online repositories. The names of the repository/repositories and accession number(s) can be found below: The University of Texas at Austin Latino Research Institute Data Repository—https://liberalarts.utexas.edu/lri/research/completed-studies/health4kids.html. Further data can be requested from the PI of the parent study, D. Parra-Medina at deborah.parra-medina@cuanschutz.edu.
